# The English and Chinese language versions of the Short Form 12-item Health Survey are equivalent

**DOI:** 10.1186/s12955-020-01653-0

**Published:** 2021-01-07

**Authors:** Daniel Y. T. Fong, Janet Y. H. Wong, Edmond P. H. Choi, K. F. Lam, C. Kwok

**Affiliations:** 1 School of Nursing, Li Ka Shing Faculty of Medicine, The University of Hong Kong, William MW Mong Block, 21 Sassoon Road, Pokfulam, Hong Kong; 2 Department of Statistics and Actuarial Science, The University of Hong Kong, Run Run Shaw Building, Pokfulam, Hong Kong; 3 School of Nursing and Midwifery, Western Sydney University, Locked Bag 1797, Penrith, NSW 2751 Australia

**Keywords:** Measurement invariance, Confirmatory factor analysis, Equivalence range

## Abstract

**Background:**

The Short Form 12-item Health Survey (SF-12v2) was originally developed in English, but it is also available in Hong Kong (HK) Chinese. While both language versions had their measurement properties well assessed in their respective populations, their measurement invariance in scores has not been examined. Therefore, we aimed to assess their measurement invariance.

**Methods:**

We conducted a cross-sectional study on individuals aged 18 years or older at a university campus. Those who were bilingual in English and Chinese were randomly assigned to self-complete either the standard English or the HK Chinese SF-12v2. Measurement invariance of the two components and eight scales of the SF-12v2 was concluded if the corresponding 90% confidence interval (CI) for the difference between the two language versions entirely fell within the minimal clinically important difference of ± 3 units. Multiple-group confirmatory factor analysis (CFA) was also performed.

**Results:**

A total of 1013 participants completed the SF-12v2 (496 in English and 517 in HK Chinese), with a mean age of 22 years (Range 18–58), and 626 participants (62%) were female. There were no significant differences in demographics. Only the physical and mental components and the mental health (MH) scale had their 90% CIs (0.21 to 1.61, − 1.00 to 0.98, and − 0.86 to 2.84, respectively) completely fall within the ± 3 units. The multiple-group CFA showed partial strict invariance.

**Conclusions:**

The English and HK Chinese versions of the SF-12v2 can be used in studies with their two components and MH scores pooled in the analysis.

## Background

Self-reported outcomes have been commonly used in clinical studies for assessing the health status or well-being of individuals. These days, most countries are multicultural with a diverse population comprising different ethnic or language groups. Conducting large-scale epidemiological studies in these places or across countries requires using instruments in different languages. Although each language version may have undergone rigorous linguistic and psychometric evaluation in its corresponding population, the resulting scores from the different language versions may not be directly comparable for at least two reasons. First, there may be potential differences in the understanding of items across different languages. For instance, the scores of one-third of the domains and facets of the WHOQOL-BREF were found to be different between the English and Hindi versions in individuals bilingual to the two languages [1]. Second, the corresponding population norms may also be different. In general, under the Universalist model of equivalence, there are six types of cross-cultural equivalence to be assessed before concluding an instrument is cross-culturally valid [2]. They are conceptual equivalence, item equivalence, semantic equivalence, operational equivalence, measurement equivalence, and functional equivalence [2, 3]. Conceptual equivalence refers to the same scale structure across the different language versions, whereas item equivalence and semantic equivalence refers to the same appropriateness and meaning, respectively, of individual items. Operational equivalence concerns with whether the instrument format, mode of administration and measurement methods are equally appropriate across the different language versions. Measurement equivalence, or also called measurement invariance, assesses the comparability of measurement properties of different language versions of an instrument. It is also related to conceptual equivalence when it comes to the comparison of construct validity. Functional equivalence is defined as the extent to which the different language versions measure equally well in what they should be measuring. It involves collective appraisal of other types of equivalence in the context of application.

The Medical Outcome Survey’s (MOS) Short-Form 36-item Health Survey (SF-36) has been a widely used health-related quality of life instrument applicable to the general population [4]. It comprises eight scales in two component summary scales—physical and mental [5]. A shorter form 12-item version SF-12 was developed to resemble the two component summary scales of the full version. To improve the discrimination and consistency of format, it was further revised to SF-12v2 by increasing the number of response options of four items from 2 to 5, and reduce that of two items from 6 to 5 [6]. The SF-12v2 is available in 148 languages, including Chinese. The measurement invariance of the SF-12v2 was assessed in 361 European-American, 187 Latina, and 107 African American postpartum women [7]. A great degree of measurement invariance was found across the three ethnic groups. However, the study involved postpartum women only and did not consider the Chinese population. Another larger-scale study assessed the construct validity of the SF-12v2 across seven ethnic groups, including 175 Chinese participants [8]. However, only the scores from the different ethnic groups were compared without any assessment of measurement invariance. Yet, the English and Chinese versions of the SF-12v2 were used in the same multi-cultural study [9].

Both the English and the Chinese versions of the SF-12v2 have had their psychometric properties well evaluated in the corresponding language groups. However, the measurement invariance and the extent to which the standard English and Chinese versions of the SF-12v2 are different have not been assessed. Measurement invariance of a self-reported instrument has been traditionally assessed by multiple-group confirmatory factor analysis (CFA) [10]. If a self-reported instrument demonstrated configural, metric, scalar, and strict invariance, the instrument could be considered measurement invariant, and the scores from different groups could be compared as if they were obtained from the same language version [11]. Alternatively, item response theory (IRT) has also been proposed to assess measurement invariance by differential item functioning [12]. The two approaches have different assumptions and provide different information on measurement invariance. IRT methods provide more information at the item response level, i.e. the difficulty parameters; whereas multiple-group CFA provides information on the relationship between latent factors and thus has been recommended when examining the equivalence of a multifactorial framework [12]. Nevertheless, in either approach, the involvement of clinical significance is limited. An instrument may have its scores differ across different language groups by only an insignificant, small difference, but it still may not attain invariance, especially when the χ^2^ statistic that is sensitive to sample size is used. On the other hand, a scale showing invariance in multiple-group CFA may still exhibit a discernible difference.

Hence, this study aimed to assess the measurement invariance of the English and Chinese versions of SF12v2, involving both statistical and clinical assessments.

## Methods

### Design

This was a cross-sectional study of Hong Kong (HK) Chinese adults who were bilingual in both English and Chinese. They were randomized to self-complete either the English or the HK Chinese version of the SF12v2, while all of them provided their demographic information. All participants consented to participate in the study before they completed the questionnaires. The study protocol and informed consent form have been approved by the local institutional review board (IRB reference number: UW13-423).

### Subjects

We included subjects who were at least 18 years old and were able to read both English and Chinese. They were recruited by advertising the study in a local institution via posters and emails. In addition, we had also set up recruitment booths at the university campus to facilitate study participation.

There was no definite sample-size requirement for testing the measurement invariance. However, a simulation has shown that the sample size of 400 per group would yield 50 to 100% power of a measurement invariance test [13]. Hence, we planned to recruit 1000 subjects who were bilingual in English and Chinese to have 500 subjects in each language group.

### Measurements and procedures

We used both the English and HK Chinese versions of the standard SF-12v2 that was composed of 12 items. The SF-12v2 provides a physical component score (PCS) and a mental component score (MCS) and scale scores for physical function (PF, 2 items), role physical (RP, 2 items), bodily pain (BP, 1 item), general health (GH, 1 item), vitality (VT, 1 item), social function (SF, 1 item), role emotion (RE, 2 items), and mental health (MH, 2 items). The SF-12v2 and the permission to use it were obtained from Optum (URL: optum.com). We also assessed the participants’ demographics, including age, gender, marital status, employment, and medical history.

The questionnaire was administered both online and in paper form. In either mode, an eligible subject was first asked for their language preference as either English, Chinese, or bilingual. Those who indicated that they were bilingual were randomly given either the English or the Chinese version of the questionnaire on a 1:1 ratio by computer and a preset randomization list for the online and paper administration modes, respectively.

### Statistical analysis

The analysis included subjects who were bilingual in English and Chinese. The scores of the eight scales and the two US norm-based components from the two language versions of the standard SF-12v2 were obtained as instructed in the scoring manual [14]. Briefly, the items were first re-coded or re-calibrated before the score of a scale was obtained as the simple algebraic sum of the corresponding item(s) standardized on the 0–100 scale. The two component scores were obtained as weighted sums of all score scores standardized by the US norms. The PCS was primarily indicated by PF, RP, BP and GH and thus weighted more on these scales, while MCS was primarily indicated by VT, SF, RE and MH and had higher weights on them.

To assess the measurement invariance of the SF-12v2 across the two languages, we first assessed the two-factor structure of the SF-12v2 by performing a confirmatory factor analysis (CFA) of participants who completed all the items of the SF-12v2 [15]. The diagonally weighted least square estimation method that accounted for the ordinal nature of the items was used to obtain the fit indices, including the root mean square error of approximation (RMSEA), the standardized root mean square residual (SRMR), the comparative fit index (CFI), and the Tucker–Lewis index (TLI). They covered the parsimony correction, absolute fit, and comparative fit, as recommended [16]. A model was considered of adequately good fit when RMSEA was close to 0.06 or lower, SRMR was close to 0.08 or lower, and CFI and TLI were close to 0.95 or greater [17]. Error covariances were added when the modification index was large and were identified in previous studies [7, 15].

Upon confirming the two-factor structure, we tested a series of increasingly restrictive hypotheses: configural, metric, scalar and strict invariance. Configural invariance refers to the same factor structure across groups. It was assessed by restricting the same two-factor structure across the two languages in a multiple-group CFA. When the corresponding fit indices fell within the afore-mentioned criteria, the two language versions have the same latent constructs indicated by the same set of items. Metric invariance refers to the same factor loadings across groups. It was assessed by further restricting the same factor loadings between the two language groups. A change (Δ) of 0.01 or below in RMSEA and CFI was considered of adequate invariance [18]. Attaining metric invariance suggests the constructs of the two languages have the same meaning to the subjects. Scalar invariance refers to the same level of a construct across groups. It was then by further restricting the same intercept across the two language groups and examining if both ΔRMSEA and ΔCFI < 0.01. If scalar invariance was demonstrated, the scale/component scores of the two language groups can be compared by the corresponding means. Strict invariance refers to the same item residuals across groups. It was assessed by additionally restricting equal residual variances and the Δ was assessed. Strict invariance means the same variances of the scales and components of the two language groups. If strict invariance was not demonstrated, we used modification indices to free items from the restriction and assessed the Δ again.

The language equivalence of the SF-12v2 was further clinically assessed by examining the 90% confidence intervals (CIs) for the differences of the component and scale scores between the two language groups. Measurement invariance in a component/scale was concluded if the corresponding 90% CI for the difference between the two language versions entirely fell within the minimal clinically important difference of ± 3 units [19].

The statistical analysis was conducted in R version 3.5.2 (R Foundation for Statistical Computing, Vienna, Austria) and the Statistical Analysis System (SAS) Version 9.4 (SAS Institute, Cary, NC).

## Results

A total of 1052 subjects consented to participate in the study, of which, 1013 completed the SF-12v2. Their mean age was 21.9 years (SD = 5.63, range = 18 to 58). There were 496 (49%) participants who completed the English version. Table 1 summarizes the demographics of the participants. There were no significant differences in subject characteristics between those who completed the English version and those who completed the Chinese version. There were significant differences in PF, RP, SF, RE, and PCS of the SF-12v2. For the English version, the Cronbach’s alpha and McDonald’s omega of PCS were 0.72 (95% CI 0.68 to 0.75) and 0.75 (95% CI 0.71, 0.78), respectively, while those of MCS were 0.83 (95% CI = 0.81 to 0.86) and 0.84 (95% CI 0.81 to 0.86), respectively. For the Chinese version, the alpha and omega of PCS were 0.68 (95% CI 0.64 to 0.72) and 0.72 (95% CI 0.69 to 0.75), respectively, while those of MCS were 0.82 (95% CI 0.80 to 0.85) and 0.83 (95% CI 0.80 to 0.85), respectively.

**Table 1:** Characteristics of 1013 bilingual participants

	English (n = 496)	Chinese (n = 517)	*p* value
	N (%) or mean ± SD	N (%) or mean ± SD	
Age (years)	21.9 ± 5.9	21.8 ± 5.4	0.869
Gender (1 missing)			0.690
Male	186 (37.5%)	200 (38.8%)	
Female	310 (62.5%)	316 (61.2%)	
Marital status (1 missing)			0.968
Single	461 (92.9%)	482 (93.4%)	
Cohabit	2 (0.4%)	2 (0.4%)	
Married	31 (6.3%)	31 (6.0%)	
Widow	1 (0.2%)	1 (0.2%)	
Divorced	1 (0.2%)	0 (0%)	
Employment (1 missing)			
Student	410 (82.8%)	432 (83.6%)	0.337
Full-time work	72 (14.5%)	80 (15.5%)	
Part-time work	6 (1.2%)	1 (0.2%)	
Job seeking	5 (1.0%)	3 (0.6%)	
Retired	2 (0.4%)	1 (0.2%)	
Medical history (5 missing)			
No	438 (88.8%)	436 (84.7%)	0.314
Yes^a^	55 (11.2%)	79 (15.3%)	
SF-12v2			
PF (2 missing)	87.6 ± 22.3	93.5 ± 15.8	< 0.001
RP (1 missing)	75.1 ± 22.3	78.0 ± 21.9	0.037
BP (1 missing)	79.0 ± 24.5	76.2 ± 21.8	0.056
GH (1 missing)	58.5 ± 23.6	57.6 ± 25.1	0.600
VT (1 missing)	53.0 ± 21.4	51.5 ± 23.7	0.297
SF (1 missing)	70.6 ± 23.0	81.0 ± 20.2	< 0.001
RE (2 missing)	68.4 ± 23.4	64.5 ± 23.8	0.008
MH (0 missing)	61.8 ± 17.5	62.8 ± 18.3	0.378
PCS (9 missing)	50.9 ± 6.9	51.8 ± 6.4	0.030
MCS (9 missing)	42.8 ± 9.4	42.8 ± 9.7	0.982

*PF* physical function, *RP* role physical, *BP* bodily pain, *GH* general health, *VT* vitality, *SF* social function, *RE* role emotion, *MH* mental health, *PCS* physical component score, *MCS* mental component score

^a^Diabetes Mellitus, Gastrointestinal diseases, Heart diseases, Hypertension, Kidney or renal diseases, Liver diseases, Respiratory diseases, Stoke, others

A total of 1004 participants completed all items on the SF12v2. They were included in the evaluation of measurement invariance. The two-factor model of the SF-12v2 showed satisfactory fit, after adding error covariances in the item pairs: Q2a–Q2b, Q3a–Q3b, Q4a–Q4b, Q6b–Q6b and Q1–Q6b that were also identified in previous studies to have higher associations than other items. The corresponding values of the fit indices were RMSEA = 0.056, SRMR = 0.052, CFI = 0.99, and TLI = 0.99, with degrees of freedom (*df*) = 48.

After restricting the same factor structure across the two language groups, configural invariance was shown with *df* = 96, RMSEA = 0.062, SRMR = 0.044, CFI = 0.96, and TLI = 0.94. Table 2 summarizes the assessments of the higher levels of measurement invariance. Metric invariance was demonstrated after further restriction of equal factor loadings, with both ΔRMSEA and ΔCFI < 0.01. However, further restricting the equal intercepts resulted in both ΔRMSEA and ΔCFI > 0.01. Hence, full scalar invariance was not shown. Large modification indices were found in Q2b, Q3b, Q4b, and Q7, which belonged to the PF, RP, RE, and SF scales, respectively. Freeing the corresponding intercepts showed partial scalar invariance with both ΔRMSEA and ΔCFI < 0.01. Further restriction on equal residual variances resulted in changes in RMSEA and CFI < 0.01, showing partial strict invariance.

**Table 2:** Measurement invariance of the two-factor structure of the SF-12v2

Model	Level of measurement invariance	Degrees of freedom	χ^2^ statistic	RMSEA	CFI	ΔRMSEA	ΔCFI
M1: Same factor pattern		96	283.5	0.062	0.96	–	–
M2: Same factor loadings, versus M1	Metric invariance	106	327.5	0.065	0.95	0.003	0.01
M3: Same factor loadings, intercepts, versus M2	Scalar invariance	116	513.4	0.083	0.91	0.018	0.04
M4: Same factor loadings, intercepts (partial),^a^ versus M2	Partial scalar invariance	112	366.8	0.067	0.94	0.002	0.01
M5: Same factor loadings, intercepts (partial),^a^ residual variances, versus M4	Partial strict invariance	124	446.8	0.072	0.93	0.005	0.01

*RMSEA* root mean square error of approximation, *CFI* comparative fit index

^a^After freeing the intercepts for items Q2b, Q3b, Q4b and Q7

**Fig. 1:**
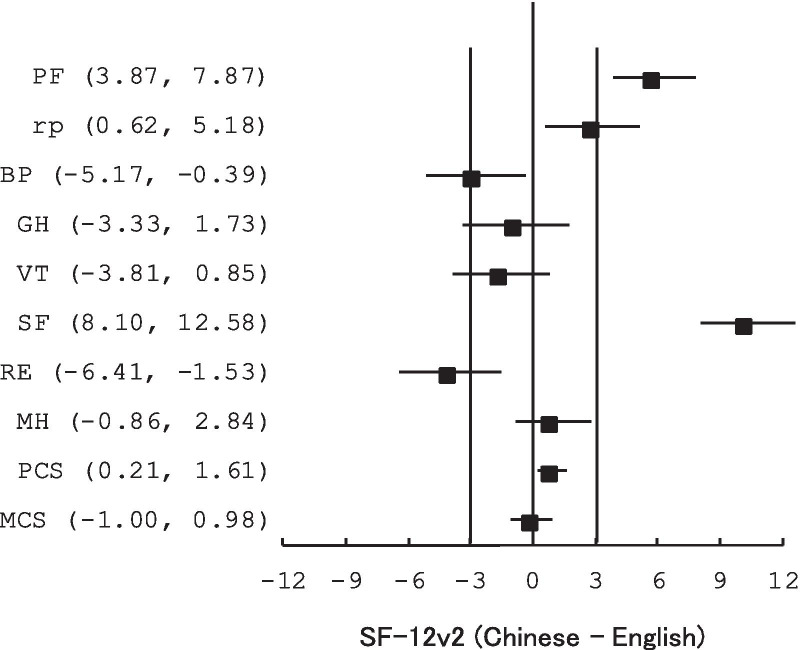
Language equivalence of SF-12v2. The error bars are the 90% confidence intervals for the differences between the two language groups

Figure 1 shows the 90% CI for the differences of the SF-12v2 components and scales between the two language groups. The PCS and MCS had their 90% CIs (0.21 to 1.61 and − 1.00 to 0.98, respectively) fall within the equivalence range of ± 3. However, only the MH out of the eight scales had their 95% CIs fall within the equivalence range.

## Discussion

This is the first study that randomized a group of subjects who were bilingual in English and Chinese to assess the measurement invariance of the SF-12v2 between the two languages. In Hong Kong, although the native language is Chinese, English is a compulsory subject in primary and secondary schools and the medium of instruction at tertiary institutions. In 2011, 77% of the population had been educated at least in a secondary school in English and should be able to read common English [20]. Hence, many people in Hong Kong can read both English and Chinese. The SF-12v2 would be applicable to them depending on their language preference.

The physical and mental components of the SF-12v2 demonstrated very good measurement invariance with the corresponding confidence intervals falling well entirely within the equivalence range of ± 3. The MH scale also had its 90% confidence interval for the language difference fall within the range. The other scales did not show measurement invariance. In particular, the PF, SF, and RE scales showed a clear violation of measurement invariance. Indeed, large modification indices were observed in Q2b, Q3b, Q4b, and Q7 that belong to the PF, RP, RE, and SF scales, respectively. The violation of measurement invariance in generally all the SF-12v2 scales is likely due to only one to two items per scale making the scales more vulnerable to the effect of differential functioning items.

The SF-12v2 showed only partial strict invariance. Strict invariance is the highest level of measurement invariance that requires the equivalence of residual variance or measurement error across groups and is rarely attained in practice [21]. Indeed, strict invariance is often not taken as a pre-requisite for testing about the mean differences [11], while some suggested lack of strict invariance may lead to bias comparison [22]. To offer a more complete picture of the level of language equivalence of the SF-12v2, we also examined the strict invariance. Nevertheless, a literature review showed partial measurement invariance was concluded in one-third of the test [11]. In general, the statistical and conceptual consequences of partial measurement invariance are not well understood [11]. There appears to be no standard on the acceptable proportion of invariant items, despite the fact that certainly the higher the better. Nevertheless, it has been suggested that ideally more than half of the items should be invariant before the corresponding scale would be considered as invariant [23]. Among the four noninvariant items of the SF-12v2, two came from the physical component and the other two from the mental component. Hence, there were 67% invariant items in both the physical and mental components. However, the single-item SF scale that included the noninvariant item Q7 is the only one that would not be considered invariant. The PF, RP, and RE scales, each composed of only one invariant item out of two, would also not be considered invariant. These are also reflected in the between-group comparisons. On the other hand, the BP, GH, VT, and MH scales would be considered invariant using the same suggested rule in the multiple-group CFA, but not in the between-group comparisons, except for the MH scale, which fell within the equivalence range. Incorporating clinical assessment by the equivalence range would add to the assessment of measurement invariance.

The use of equivalence range for establishing measurement invariance has also been adopted in previous studies [24, 25]. The English and Chinese versions of the self-reported questionnaires the Functional Assessment of Cancer Therapy-General (FACT-G), the European Organization for Research and Treatment of Cancer Core Quality of Life Questionnaire (EORTC QLQ-C30), and the Repeatable Battery for the Assessment of Neuropsychological Status (RBANS) were shown to be generally equivalent by using the equivalence ranges defined as ± 0.25 standard deviation (SD) that corresponds to a small Cohen effect size. We chose to use the previously reported minimally important difference of the SF-12v2 to ensure direct comparison with the reported minimally important difference. Nevertheless, the two approaches gave essentially the same conclusions in our application. However, the use of randomization in our study avoids the need of adjustment for other between-group differences as in the previous studies.

A cross-over design may be more appealing for demonstrating equivalence, since each individual serves as his or her own control, and it requires a smaller sample size. We adopted a between-subject randomized design to minimize the burden of administering a long questionnaire and participants’ possible annoyance in responding to questions of the same content although in different languages. Moreover, the wash-out period would need to be long enough to avoid recall of the first response, but it would increase the administrative difficulty as participants would be unlikely to wait long before completing the questionnaire again. On the other hand, our target of bilingual individuals should have resulted in a relatively younger sample with an average age of around 22 years, despite our sample included individuals as old as 58 years. Since the use of multiple-group CFA is sample dependent, our results may not be generalized to the older group and further studies are desirable. Furthermore, we have not evaluated the criterion validity of the SF-12v2 by comparing it against the full version SF-36. However, given both the English and Chinese versions of SF-12v2 have been well tested against the corresponding SF-36 [5, 6], the potential influence of the concern of criterion validity in our comparisons would be minimal. Finally, we have not assessed the measurement invariance of the two language versions when they are used in their corresponding cultural groups. Further studies for assessing the possible cultural influences would be desirable.

## Conclusions

The physical and mental component scores of the SF-12v2 demonstrated high measurement invariance between the English and Chinese versions. These tools can be used in multilingual studies for assessing the physical and mental components of the quality of life in adults.

## Data Availability

The datasets generated and analyzed during the current study are not publicly available to preserve the privacy of the participants but are available from the corresponding author on reasonable request.

## Abbreviations

BP: Bodily pain
CFA: Confirmatory factor analysis
CI: Confidence interval
GH: General health
HK: Hong Kong
MOS: Medical Outcome Survey
MCS: Mental component score
MH: Mental health
PCS: Physical component score
PF: Physical function
RE: Role emotion
RP: Role physical
SD: Standard deviation
SF: Social function
SF-12: Short-Form 12-Item Health Survey
VT: Vitality
